# Improving eye care for veterans with diabetes: An example of using the QUERI steps to move from evidence to implementation: QUERI Series

**DOI:** 10.1186/1748-5908-3-18

**Published:** 2008-03-19

**Authors:** Sarah L Krein, Steven J Bernstein, Carol E Fletcher, Fatima Makki, Caroline L Goldzweig, Brook Watts, Sandeep Vijan, Rodney A Hayward

**Affiliations:** 1 Health Services Research and Development, VA Ann Arbor Healthcare System, Ann Arbor, Michigan, USA; 2 Department of Internal Medicine, University of Michigan, Ann Arbor, Michigan, USA; 3 General Internal Medicine and Clinical Informatics, VA Greater Los Angeles Healthcare System, Los Angeles, California, USA; 4 Louis Stokes Cleveland VA Medical Center, Cleveland, Ohio, USA

## Abstract

**Background:**

Despite being a critical part of improving healthcare quality, little is known about how best to move important research findings into clinical practice. To address this issue, the Department of Veterans Affairs (VA) developed the Quality Enhancement Research Initiative (QUERI), which provides a framework, a supportive structure, and resources to promote the more rapid implementation of evidence into practice.

**Methods:**

This paper uses a practical example to demonstrate the use of the six-step QUERI process, which was developed as part of QUERI and provides a systematic approach for moving along the research to practice pipeline. Specifically, we describe a series of projects using the six-step framework to illustrate how this process guided work by the Diabetes Mellitus QUERI (DM-QUERI) Center to assess and improve eye care for veterans with diabetes.

**Results:**

Within a relatively short time, DM-QUERI identified a high-priority issue, developed evidence to support a change in the diabetes eye screening performance measure, and identified a gap in quality of care. A prototype scheduling system to address gaps in screening and follow-up also was tested as part of an implementation project. We did not succeed in developing a fully functional pro-active scheduling system. This work did, however, provide important information to help us further understand patients' risk status, gaps in follow-up at participating eye clinics, specific considerations for additional implementation work in the area of proactive scheduling, and contributed to a change in the prevailing diabetes eye care performance measure.

**Conclusion:**

Work by DM-QUERI to promote changes in the delivery of eye care services for veterans with diabetes demonstrates the value of the QUERI process in facilitating the more rapid implementation of evidence into practice. However, our experience with using the QUERI process also highlights certain challenges, including those related to the hybrid nature of the research-operations partnership as a mechanism for promoting rapid, system-wide implementation of important research findings. In addition, this paper suggests a number of important considerations for future implementation work, both in the area of pro-active scheduling interventions, as well as for implementation science in general.

## Background

The need to more rapidly move important research findings into clinical practice is recognized as a critical part of closing the quality chasm [1,2]. Often, the transition from research breakthrough to clinical practice takes many years and progresses haphazardly due to fragmentation in funding, a lack of partnerships and no consistent framework or incentives to encourage movement along the research to practice pipeline [3]. Moreover, quality gaps can occur due to a number of "translation blocks" [3,4], including a potential block in actual implementation that historically has received little attention from the research community or research funding agencies. To address these issues, the Department of Veterans Affairs (VA) developed the Quality Enhancement Research Initiative (QUERI), which provides tools as well as a supportive structure and resources to promote the rapid implementation of evidence into practice [5].

This article is one in a *Series *of articles documenting implementation science frameworks and approaches developed by the U.S. Department of Veterans Affairs (VA) Quality Enhancement Research Initiative. QUERI is briefly outlined in Table 1 and is described in more detail in previous publications [6,7]. The *Series*' introductory article [8] highlights aspects of QUERI that are related specifically to implementation science, and describes additional types of articles contained in the *QUERI Series*. The Diabetes Mellitis QUERI (DM-QUERI) is one of the current QUERI Centers, and is one of the original eight Centers established in 1998 [5,9]. Type 2 diabetes affects nearly 20% of veterans who use the VA health care system, or more than one million veterans at any given time. Not only is diabetes a prevalent condition, it is also associated with substantial morbidity, mortality, and increased healthcare costs [10-13]. Among people with diabetes, the presence of specific risk factors, such as persistently elevated glucose levels and poorly controlled hypertension, can lead to severe and devastating complications including end-stage renal disease, amputation and blindness. Further, up to 80% of patients with diabetes will develop or die from macrovascular disease, such as heart attack and stroke [14,15]. Reducing preventable morbidity and mortality among veterans with diabetes is the primary objective of DM-QUERI, with specific diabetes-related priority areas that include: 1) optimizing management of cardiovascular risk factors; 2) decreasing rates of diabetes-related complications, including visual loss, kidney disease, and lower-extremity ulcers and amputation; 3) improving patient self-management; 4) better management of patients with diabetes and other chronic comorbid conditions; and 5) advancing clinically-meaningful quality/performance measurement as an important tool for promoting and assessing quality improvement interventions. Examples of work by DM-QUERI that address these different priority areas can be found in prior publications [16-19]

**Table 1:** The VA Quality Enhancement Research Initiative (QUERI)

The U.S. Department of Veterans Affairs' (VA) Quality Enhancement Research Initiative (QUERI) was launched in 1998. QUERI was designed to harness VA's health services research expertise and resources in an ongoing system-wide effort to improve the performance of the VA healthcare system and, thus, quality of care for veterans.
QUERI researchers collaborate with VA policy and practice leaders, clinicians, and operations staff to implement appropriate evidence-based practices into routine clinical care. They work within distinct disease- or condition-specific QUERI Centers and utilize a standard six-step process:
1) Identify high-risk/high-volume diseases or problems.
2) Identify best practices.
3) Define existing practice patterns and outcomes across the VA and current variation from best practices.
4) Identify and implement interventions to promote best practices.
5) Document that best practices improve outcomes.
6) Document that outcomes are associated with improved health-related quality of life.
Within Step 4, QUERI implementation efforts generally follow a sequence of four phases to enable the refinement and spread of effective and sustainable implementation programs across multiple VA medical centers and clinics. The phases include:
1) Single site pilot,
2) Small scale, multi-site implementation trial,
3) Large scale, multi-region implementation trial, and
4) System-wide rollout.
Researchers employ additional QUERI frameworks and tools, as highlighted in this *Series*, to enhance achievement of each project's quality improvement and implementation science goals.

In this paper we illustrate the use of the QUERI six-step process (Table 1) as a framework for improving the delivery of VA eye care services for veterans with diabetes. Specifically, we describe an integrated series of projects, guided by the QUERI process, which progressed from identifying a high-priority condition to an implementation intervention in approximately five years. The importance of a funding mechanism to support QUERI projects, including implementation work, also is discussed. We identify several important considerations for future implementation work, both specific to proactive scheduling and in general, as well as some challenges with the QUERI process. The information provided in this paper is intended to help inform researchers, policymakers and managers who might be studying or engaged in implementing research into practice.

## Methods

### Using the QUERI Steps to improve eye care for veterans with diabetes

Although work by the diabetes QUERI is multi-faceted, preventing diabetes-related visual loss is a specified area of concern. As depicted in the QUERI six-step process, implementation is part of a continuum or pipeline that progresses from identifying high priority conditions/populations to determining evidence-based practices and quality gaps to designing, implementing and evaluating quality improvement programs. In the following sections, we describe a series of projects using the six QUERI steps to illustrate how this process guided work by DM-QUERI to assess and improve eye care for veterans with diabetes. We begin with an overview of the scope of the problem (QUERI Step 1) and then focus on specific projects for Steps 2–6, including a brief discussion of the project background, methods, results and implications, as the full results of these projects are published elsewhere [20,21]. Given the focus on implementation, we provide greater detail about the eye care implementation project (QUERI Steps 4/5/6) and end with a more general discussion and conclusion section that summarizes key considerations drawn from this body of work, as well as our experiences using the QUERI process.

### QUERI Step 1: Priority conditions/issue

Diabetes is the leading cause of new cases of blindness in adults ages 20–74 in the U.S. [22]. In the VA, approximately one-quarter of all eye procedures performed in FY1998 were for veterans with diabetes, and among patients with diabetes examined by an ophthalmologist nearly 5% were blind [23]. Providing training for blind veterans through the Blind Rehabilitation Center costs approximately $20,000–$25,000 during the first year [23], and this is only the monetary cost that does not take into account the significant impact of blindness on patient quality of life. Thus, preventing blindness among veterans with diabetes is a high-priority issue for the VA and, as part of our goal to reduce preventable morbidity and mortality among veterans with diabetes as previously described, one of several important issues for DM-QUERI.

### QUERI Step 2: Evidence-based practices

Evidence suggests that 90% of visual loss due to diabetic retinopathy can be prevented through optimal medical and ophthalmologic care, including early detection and laser therapy [24-27]. There is little disagreement that laser therapy for established diabetic retinal complications is an effective treatment. However, the costs and trade-offs of the standard recommendation to screen all diabetes patients annually to promote early detection versus tailoring screening frequency to patient need has been a topic of debate. To address this issue, a cost-utility study was conducted to examine the marginal cost-effectiveness of different screening intervals for patients with type 2 diabetes [20].

This research was conducted using simulation techniques (a Markov model) and a population of patients with diabetes based on data from the Third National Health and Nutrition Examination Survey (NHANES III) [20]. The simulation model included information about disease progression, utility estimates, mortality rates, and the relationship between glycemic control and retinopathy obtained from prior studies, such as the UK Prospective Diabetes Study [27]. Costs were estimated from the perspective of a third-party payer and were based primarily on Medicare reimbursement rates [20].

The study showed that risk of blindness varies by both age and a patient's level of glycemic control over the past 2–3 months. The patients who benefit most from annual screening and for whom it is cost-effective are those with very poor glycemic control. However, for those patients whose previous exam was normal [20], routine annual screening is not appreciably better in preventing blindness than screening every 2–3 years, and annual screening could be an unnecessary burden for some patients. Closer monitoring of those with known disease also appeared to be a key factor in preventing diabetes-related blindness.

The results of this Step 2 project, along with similar findings by other researchers [28,29], provided some of the evidence for review and discussion by a multidisciplinary panel of a proposed change in the prevailing quality standard from requiring annual screening for all patients with diabetes to a risk-stratified approach. In addition, this study helped identify lack of close follow-up as a possible quality gap that could result in preventable visual loss among patients with diabetes, thus leading to Step 3 in the QUERI process.

### QUERI Step 3: Quality/performance gaps

Eye screening is important, but screening alone does not prevent visual loss or blindness. In fact, since FY2002 retinal screening rates for VA patients with diabetes have been greater than 70% according to performance measurement reports prepared by the VA Office of Quality and Performance. To better understand the circumstances surrounding preventable visual loss among patients with diabetes, a study was undertaken that focused specifically on the timing of retinal photocoagulation (i.e., laser eye surgery) as a key issue in preventing visual loss [21].

Physician reviewers examined medical records from a university ophthalmologic center and two VA Medical Centers for 238 patients who had photocoagulation for proliferative diabetic retinopathy or macular edema. Based on pre-specified criteria [21], the reviewers identified more than 100 patients (43%) whose visual loss was considered preventable by earlier treatment. Screening-related failures accounted for approximately one-third of the cases of suboptimal timing. However, all of these failures were for patients who had gone more than three years without an exam. Not a single case of preventable visual loss was identified for patients who had gone 1–3 years without a screening exam. More importantly, two-thirds of cases were associated with problems related to surveillance of those with identified disease, including inadequate follow-up, delays in treatment scheduling, or unexpectedly rapid disease progression.

The results of this Step 3 study identified a lack of close follow-up of those with known disease as a potentially important gap in quality of care. Moreover, these findings suggested that the prevailing performance measure, which encouraged an annual exam for all patients with diabetes, could potentially decrease true quality. Trying to screen everyone annually consumes much of the eye care clinics' limited resources, thereby making it more difficult to aggressively monitor and follow veterans at highest risk of blindness [21].

### QUERI Steps 4/5/6: Implementation and evaluation of improvement program/project

With a high-priority issue identified (QUERI Step 1), evidence to support a change in the diabetes eye screening performance measure (QUERI Step 2), and an identified gap in quality of care (QUERI Step 3), the next step was implementation. Accordingly, DM-QUERI focused on two initiatives: 1) an intensive lobbying effort to revise the existing Health Plan Employer Data and Information Set (HEDIS^®^) [30] and VA performance measures for diabetes eye care, and 2) an implementation project to promote close follow-up of high-risk patients. First, as mentioned in our discussion of QUERI step 2, changing the diabetes eye care quality measure used in HEDIS^® ^and the VA's quality monitoring system was actively being debated. Efforts directed toward changing the current measurement policies began well before the eye care implementation project and continued throughout much of the study period, as described in more detail in the next section. Second, DM-QUERI received funding through VA's Health Services Research and Development Service's (HSR&D) service-directed project mechanism, which was specifically established for implementation studies, to support an eye care implementation project. The proposed implementation project was a small scale multi-site study (or phase 2 project as described in Table 1) with a quasi-experimental design. However, the design was changed to a single-site pilot (or phase 1 project as described in Table 1) because of difficulty with implementation. Institutional review board approval for this project was obtained from the participating VA medical centers.

## Results

### Implementation project design

There are many studies of interventions to improve the management of patients with diabetes [31,32]. However, given that the focus of the proposed eye care implementation project was on scheduling and follow-up, rather than diabetes care per se, we chose a conceptual design based on successful strategies used in other types of scheduling interventions [33] and a general model of organizational change as described by Gustafson et al. [34]. Specifically, it has been shown that improvements in rates of adult immunization and cancer screening are most likely to occur through organizational changes in staffing and clinical processes [33]. These changes include: (1) establishing a separate clinic devoted to screening and prevention activities, (2) using planned clinic visits for prevention, (3) using techniques similar to continuous quality improvement, and (4) delegating specific prevention responsibilities to non-physician staff. Accordingly, the planned change for the eye care project was to shift the coordination of diabetes eye care from primary care to the eye clinic, and to provide the eye clinic staff with automated tools that would facilitate the scheduling of less frequent screening exams for low-risk patients and more aggressive follow-up of veterans at higher risk.

**Figure 1:**
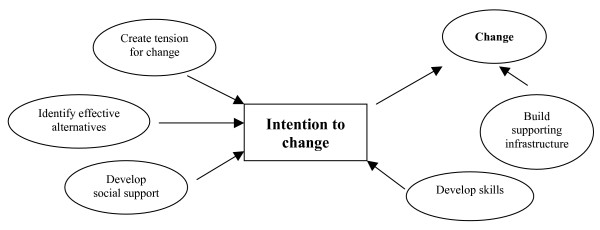
Eye Care Scheduling Intervention Implementation Framework. Based on Gustafson, et al. [34].

To help guide the implementation process [35], we employed an implementation model derived from prior work in the area of organizational and individual change (Figure 1) [34]. This model consisted of: 1) creating tension for change, 2) identifying effective alternatives, 3) developing social support, 4) developing skills, and 5) building infra-structure. During the initial stages, a major focus of the diabetes eye care project was on building the infrastructure required to support the proposed changes and improve the care of patients with diabetes.

### Building infrastructure

**Figure 2:**
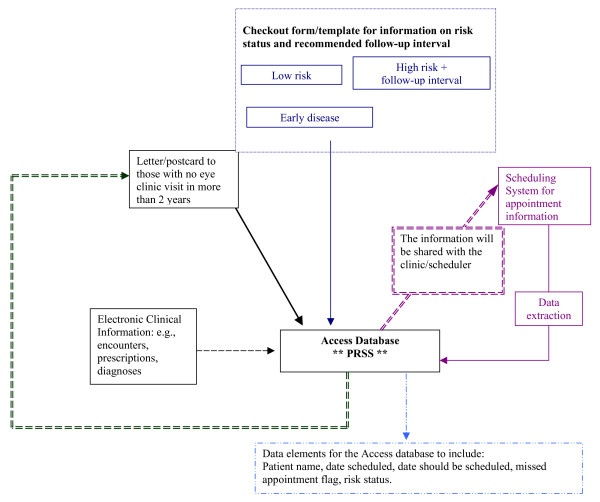
Logic Map of the Progressive Reminder and Scheduling System (PRSS).

A cornerstone of the eye care intervention was a system for automatically tracking patients based on risk status – "Progressive Reminder and Scheduling System (PRSS)" (Figure 2). The PRSS required three basic pieces of information: 1) risk status, which is assigned by the eye care provider following a clinical exam; 2) follow-up interval, which is the recommended time for the patient's next visit; and, 3) appointment status, which includes whether an appointment is scheduled, whether a visit is made, or if the appointment is cancelled or missed.

Despite the sophistication of VA's health information technology [36], only appointment status is currently available in an extractable electronic format. Risk status and recommended follow-up are generally part of the electronic health record, but are in text format only as part of the clinician's medical note. The appointment and scheduling system is distinct from the rest of the electronic health record. Consequently, a mechanism to capture patient-risk status and recommended follow-up time had to be developed along with a process for combining this information with appointment and scheduling data. Working with local information technology personnel, we tested a number of strategies for obtaining and integrating the necessary information; however, the inability to connect the scheduling system with the clinical data system prevented the development of a fully automated proactive scheduling system. So, after several months a simplified, manual version of the PRSS was developed using a Microsoft^® ^Access database. Initial development of the PRSS took place at one study site (Site A) with the intent of developing similar but organizationally tailored systems at two other study sites (Sites B and C).

The database was populated by identifying a cohort of patients with diabetes using encounter and prescription data obtained from national VA databases [37,38]. Next, in collaboration with the Site A eye clinic, an existing "check out form" was modified to collect risk status information and the recommended follow-up interval. The modified form prompted the provider (generally an ophthalmology resident) to record risk status using three risk categories: 1) low risk or normal exam, 2) early disease (e.g., micro aneurysms without macular edema), and 3) high-risk (i.e., patients with disease progression, neo-vascularization on the disc or macular edema). An "other" category was included to identify diabetes patients who might require closer follow-up due to eye problems other than retinopathy, such as glaucoma or cataracts. The provider was asked to indicate a follow-up timeframe for those patients who were identified as high risk or in the other category.

Information about risk status and recommended follow-up time from the check-out forms was entered into the Access database. Based on the number of months specified by the provider, a recommended follow-up date was calculated for those patients identified as high risk. Diabetes patients who had a normal exam, and no other condition specified, were automatically assigned a two-year follow-up appointment, while those with mild disease were assigned a one-year follow-up appointment. This information could then be merged with data from the scheduling system to identify patients with high-risk eye conditions who either were not scheduled for an appointment within the recommended time-frame or who were already past the time for their recommended appointment (e.g., 30 days past the recommended follow-up date). This information also facilitated the pro-active follow-up, by clinic staff, of those individuals at greatest risk for preventable visual loss.

In addition, the PRSS database was used to identify patients with no eye appointment in the past two years. This step was not part of the original study plan but was requested by the service-directed project review committee. After discussions with VA Ophthalmology personnel and the ambulatory care service leadership at Site A, it was decided to send a letter to individuals with no identified appointment in the past two years, signed by the Associate Chief Of Staff for primary care. Along with the letter, veterans received a brief questionnaire asking them to indicate whether they received their eye care outside the VA system or at another VA facility and, if not, whether they would like to be contacted so that a visit could be scheduled.

### Creating tension for change and identifying effective alternatives

In conjunction with creating a supportive technology infrastructure, efforts to address other factors, as identified in our implementation framework (i.e., gaining an understanding of the current climate and operational structure of the eye care clinic(s) at each study site) also were underway. More than 80 eye clinic personnel including attending physicians, residents, nurses, technicians, and clerks completed a mailed survey, and approximately 45 participated in semi-structured interviews. Information collected as part of the survey and interviews focused on the perceived adequacy of clinic resources, job satisfaction, clinic goals, functional issues, and suggestions for improvement. Subsequently, this information was used to identify how the PRSS might be tailored to function at each site. It also was used to provide a platform for discussing the potential advantages or disadvantages of the proposed changes relative to the current system with key persons in the organization (i.e., identify effective alternatives and create tension for change).

Creating tension for change and identifying effective alternatives required becoming more actively involved in the policy arena. Not only were researchers involved by producing evidence (as described in Step 2 above), they also served as technical experts while policy discussions about changing the diabetes eye care performance measure were in progress. In particular, DM-QUERI – through research publications and direct representation on the National Diabetes Quality Improvement Alliance – strongly advocated revising the diabetes eye care quality measure used in HEDIS^® ^and the VA's quality monitoring/performance measurement system. Although the VA/Department of Defense (DoD) diabetes guidelines already included a risk-stratified approach for diabetes eye care based on insulin use, level of glycemic control, and risk status, the approach was difficult to implement as a performance measure. The proposed alternative measure simply advocated for every-other-year eye exams for patients at low risk, and continued, annual or more frequent exams for patients at high risk.

### Developing social support and skills

Understanding context is important but not sufficient when implementing changes in a clinical setting [3]. The research team made a concerted effort to establish the support of both administrative and clinical leaders at each of the study sites for the proposed restructuring in the delivery of diabetes eye care. Developing skills involved training residents and other clinic staff in how to use the modified checkout forms, as well as revising the forms based on feedback from clinic personnel. Periodic reinforcement also was provided to encourage the continuing use of the forms.

### Implementation project results

#### Building infrastructure

The initial PRSS database at study site A contained a cohort of approximately 5,500 unique veterans with diabetes. From November 2004 through June 2005 more than 780 checkout forms were completed during an eye-care visit with a diabetes patient at study site A. The provider assigned risk status for these visits as shown in Table 2. A subset of patients was subsequently selected for testing the follow-up component of the PRSS. Appointment information was extracted for this subset to determine the status of those in the high-risk group, such as whether a visit was made within the recommended timeframe. The plan was for the eye clinic to use this information to initiate pro-active follow-up of those with missed appointments. For several reasons, including resource constraints and the end of study funding, this part of the system never became operational.

**Table 2:** Provider Assigned Risk Status Based on Check out Forms Completed at Site A From November 2004 – June 2005

**Risk Status**	**All Check out Forms (N = 783) % (n)**
Normal Exam	44 (345)
Early Disease	19 (146)
High Risk	15 (114)
Other*	20 (154)
Missing	3 (24)

* Other were generally patients requiring closer follow-up for conditions such as glaucoma or cataracts as well as patients who were scheduled for return visits following laser therapy or some other type of eye procedure.

More than 2300 patients with no identified eye-care visit in the past two years were identified using the site A PRSS database. Approximately 60% (1375) completed the mailed survey that showed that 952 (69%) patients were receiving eye care services elsewhere (Table 3). However, 305 (22%) patients without another eye care provider expressed an interest in having an appointment scheduled. Unfortunately, fiscal and other logistical constraints precluded scheduling exams for many of these patients. This was a difficult situation for both the involved researchers and the facility administrative and clinical leaders who had to employ a VA-wide prioritization strategy to determine who would be seen immediately and who would have to be accommodated as resources allowed. As of April 2006, less than 15% of the patients who expressed interest in scheduling an appointment had been seen in the eye care clinic. However, since patients without exams were not necessarily high-risk, as defined in this paper, but of unknown risk status, getting even 15% of these individuals screened was an improvement.

**Table 3:** Patients With No Identified Eye Exam at Site A in Past 2 Years

**Survey Response**	**Respondents (N = 1375) % (n)**
Had exam at non-VA facility	58 (798)
No exam and would like to be contacted	22 (305)
Had exam at other VA facility	11 (154)
No exam but had tried to make an appointment	4 (60)
Does not have diabetes	2 (24)
Other (e.g., had exam, did not want to make appointment)	2 (34)

### Creating tension for change and identifying effective alternatives

Our surveys and interviews of eye clinic personnel produced a number of common themes. More than 80% of respondents indicated that their work was rewarding and that the care provided was "high quality." However, there also was dissatisfaction related to staffing and equipment, a stressful working environment, and feeling too busy to provide all the care that was needed. In addition, most respondents believed that patient waiting times were too long.

In 2005, after nearly five years of discussion (and near the conclusion of the implementation project) the revised performance measure allowing every other year eye exams for patients at low risk (with continued annual exams for high risk patients) was adopted by HEDIS^® ^and the VA's performance measurement system.

### Developing social support and skills

A number of meetings with administrative, clinical, and clerical personnel were conducted at all study sites throughout the study period. Revised check-out forms, which included information about risk status and recommended follow-up for patients identified as high-risk, were developed and used by eye clinic providers at two of the three study sites. The amount of support for enacting the changes needed to implement the PRSS was variable both across sites and over time. In addition, the extent to which the check-out forms were used at the two sites cannot be fully assessed due to incomplete information about the number of patients or patient visits while the forms were in use.

## Discussion

The QUERI process provides a systematic approach for moving along the research to practice pipeline. Guided by the six-step QUERI process, DM-QUERI conducted several research projects (QUERI steps 1–3) that, in turn, provided the basis for an implementation project. Specifically, a high-priority issue was identified, evidence to support a change in the diabetes eye screening performance measure was developed, and a quality gap was identified. Building on information generated by these studies, we undertook an implementation project to improve eye care and prevent visual loss among VA patients with diabetes. This project involved shifting responsibility for the coordination of diabetes eye care to the eye clinic and using automated tools to facilitate less frequent screening of low-risk patients and more aggressive follow-up of veterans at higher risk. We accomplished and learned much during the course of this implementation project. However, despite devoting substantially more resources to the project than were originally budgeted, we did not succeed in developing a fully functional system. In retrospect, we now appreciate the potential value of the four phases within the QUERI implementation framework (Table 1). We ambitiously proposed a small scale, multi-site implementation project before completing a single site pilot. Ideally, having conducted the pilot work, as just described, and with a more functional scheduling intervention, we would now move on to the multi-site study. Plans for this next phase may have to wait until certain technological issues our resolved. Nonetheless, this research has produced important information to help us further understand patients' risk status and potential gaps in follow-up at participating eye care clinics, as well as other general lessons for implementation science, especially as it relates to proactive, risk-stratified scheduling and follow-up.

First, even with the desire for rapid implementation, development and preliminary testing of technology-based implementation tools should not be rushed – in other words have it built or they will not come. The research team spent a considerable amount of time working with local information technology personnel on just one key aspect, which was to identify patient-risk status in a consistent, easy and interpretable fashion. Even with the VA's electronic medical record, many important clinical elements, such as the presence of retinopathy, are in text notes and not easily extractable. We identified several possible solutions to this problem, such as a progress note template that would allow for extractable data fields or modifying a health factors summary form to prompt entry of certain required information as part of the charting process. Two prototypes were created and plans were being made for testing and refinement of the electronic template in collaboration with the eye clinic staff and physicians. However, neither prototype was implemented, due in part to staff turnover and a lack of active support from the eye clinic's clinical leadership at the main development site. As a workaround, modifications were made to an existing hard copy checkout form. However, with inconsistent use by the myriad of providers rotating through the eye care clinic and with a primarily electronic medical record system, sustainability of the hard copy forms is unlikely. Moreover, as time passed any momentum or enthusiasm that may have been generated among clinic staff was soon diminished as the "promise" of a better system did not materialize.

Second, this project highlights the importance of aligning national policy with a planned change in practice, as part of creating tension for change. Our surveys and interviews suggested that many clinic staff were not entirely satisfied with current clinic operations, and key individuals at the sites agreed that the proposed changes might be beneficial for improving the delivery of eye care for patients with diabetes. Neither of these conditions was sufficient, however, to overcome the pressure exerted by the existing demands on the eye clinics, including the current performance measure, which still emphasized annual visits at the time of the project. Specifically, the research team was encouraging changes based on the evidence and an anticipated change to every-other-year exams, but due to the political nature of the negotiation process it took two years longer than expected for this change to be adopted by HEDIS^® ^and the VA. Furthermore, even with the recent adoption of every-other-year exams for those with a prior normal exam, there is still no external incentive for close follow-up of those with known retinopathy (i.e., a measure that requires that patients with known eye disease have a follow-up visit within two months of the recommended interval might be warranted).

Third, as others have found [39], garnering local support for an implementation project requires considerable effort. At one site this involved overcoming initial suspicion about why "researchers" were interested in the eye clinic. At another site, an ongoing feud between services (optometry and ophthalmology) overwhelmed any attempt to enact a change that required coordination and cooperation in care delivery. At the third site, there was clear support from facility leaders, such as the Chief of Staff, and initial agreement from the ophthalmologist who ran the clinic, but once the project started this support quickly diminished, as other issues (e.g., personnel problems) took precedence. In addition, stress on the eye clinic's resources was so severe that even if everyone agreed on the long-term benefits, any changes that required initial training and learning were unlikely to occur unless workload was reduced.

Fourth, this project suggests that in some situations to change one element you may need to change the entire system. We set out to help the eye clinic develop a proactive scheduling system for diabetes-related eye care because of a quality problem with that clinical condition. However, perhaps we should have focused on designing a more efficient, proactive scheduling system that would apply to all patients seen in the clinic, not just for veterans with diabetes. This issue began to emerge during our discussions with clinic staff, but the reasons for pursuing such a strategy are even more apparent after the fact. In particular, most everyone is interested in a more efficient and effective scheduling system, so you develop common ground even with those that may not care about diabetes eye exams, specifically. It also is easier to get people to change the general procedures for every case than it is to get them to use a new system for patients with diabetes, while using the old system for other cases. In fact, as we learned, approximately 20% of eye-care visits for people with diabetes are not related to diabetes eye disease, thus making a system for diabetic eye disease even more confusing.

## Conclusion

Moving important research findings into clinical practice to ensure the efficient and effective delivery of healthcare services is important for improving healthcare quality. This article provides an example of how the VA QUERI program, specifically the QUERI six-step process and dedicated funding support through VA/HSR&D's service-directed project mechanism, facilitated a fairly rapid progression from developing evidence to inform the eye care screening debate – to identifying quality gaps related to close follow-up of high-risk patients – to implementation of a quality improvement intervention that addresses both eye screening and follow-up for patients with diabetes. While it is not possible to know how this series of activities might have progressed without QUERI, it seems doubtful that such an integrated set of projects could have been conducted in an approximately five-year timeframe without the process and structural support of the QUERI program. Moreover, without a specified funding mechanism for implementation work, it is unlikely that our work in this area would have progressed beyond Step 3 or identifying the quality gap.

The eye care implementation project was essential for the collection of important data to further characterize the risk status of veterans with diabetes who receive eye care services in the VA, and to better examine the extent to which there may be problems with close follow-up of high-risk patients. In addition, a revised diabetes eye care performance measure was adopted inside and outside VA, and we developed a prototype, proactive, risk-stratified system that can be used to support and inform future ongoing work in this area. Our experience with the eye care implementation project also has provided further insight into the implementation of scheduling interventions including issues related to time, project scope, and the importance of aligning policy with practice.

However, even with the support provided by QUERI a feasible and sustainable change in the delivery of eye care services for veterans with diabetes is yet to be accomplished. In addition to the project specific issues just discussed, we would like to highlight a few other issues that affected our work and deserve special consideration for advancing the field of implementation science. While implementation research is an integral part of improving healthcare, there are certain constraints associated with the hybrid nature of this type of research and operations enterprise that need to be addressed. In particular, both funding and timeline requirements for conducting implementation studies must be sufficient and flexible enough to support the scope of the project.

Funding and operational issues precluded our use of project funds to pay facility information technology staff for time they devoted to the project. Moreover, project funds were not available until the research requirement of obtaining institutional review board approval was completed, which caused significant delays. Obtaining institutional review board approval also required significant staff time and resources since the project had to be reviewed and approved at each participating site. Moreover, one of the original project sites was eventually excluded from the study and replaced with another site because of difficulties associated with trying to meet the specific human subjects' requirements at the original site. Resource constraints at the facility level also posed a substantial problem in our attempt to facilitate follow-up of patients with no visit in the past two years, which had actually been included in the project at the request of the scientific review board that approved the project for funding. Finally, despite the importance of learning by doing we also must be cognizant of the potential adverse consequences of unsuccessful implementation efforts, which for this project included not meeting the expectations or needs of both providers and patients, as we do not want to jeopardize future initiatives that could lead to significant improvements in patient care and outcomes.

In conclusion, we believe that work by DM-QUERI to promote changes in the delivery of eye care services for veterans with diabetes demonstrates the promise of the QUERI process in facilitating the more rapid implementation of evidence into practice. There remain many challenges for those engaged in implementation work; however, by continuing to share our experiences we can overcome many current "implementation blocks." Such active learning is already underway within the QUERI program, as evidenced by the continuing progress of QUERI with rolling out a major regional demonstration project for collaborative care, and also is likely to benefit others as the implementation imperative continues to take hold.

## Competing interests

The author(s) declare that they have no competing interests.

## Authors' contributions

SLK participated in the conduct of the implementation project, data acquisition and analysis, and she wrote the manuscript. SJB participated in the design and conduct of the implementation project and assisted in writing the manuscript. CEF participated in the conduct of the implementation project, data acquisition, and writing the manuscript. FM participated in the conduct of the quality gaps and implementation projects, data acquisition, and writing the manuscript. CLG participated in the conduct of the implementation project and writing the manuscript. BW participated in the conduct of the implementation project and writing the manuscript. SV conducted the cost-utility project and participated in writing the manuscript. RAH conducted the quality gaps project, participated in the conduct and design of the cost-utility and implementation projects, and participated in writing the manuscript. All authors read and approved the final manuscript.
